# Ecological Drivers of Molt‐Breeding Overlap, an Unusual Life‐History Strategy of Small‐Island Birds?

**DOI:** 10.1002/ece3.70607

**Published:** 2025-01-16

**Authors:** Christopher C. De Ruyck, Nicola Koper

**Affiliations:** ^1^ Natural Resources Institute University of Manitoba Winnipeg Manitoba Canada

**Keywords:** birds, Caribbean, ecological release, island biogeography, life‐cycle phenology

## Abstract

Terrestrial bird populations on small, species depauperate islands often experience selection for generalist foraging traits via ecological release; however, it is unclear how island conditions may uniquely influence other life‐history characteristics of small‐island birds, such as the unusually high rates of molt‐breeding overlap exhibited on the island of Grenada. To explore this question, we collected data on the life cycles and diets of 10 commonly occurring Grenadian bird species to assess the degree of generalist foraging and evaluate how seasonal patterns in diet niche breadth and diet overlap among species relates to the high rates of molt‐breeding overlap. We evaluated three hypotheses explaining drivers of molt‐breeding overlap (constraints on molt rate, unpredictable food abundance, and limited duration of food abundance), and suggest that widespread overlap in small‐island tropical communities may be the result of generalist foraging adaptations and restricted time periods of sufficient invertebrate availability for successful breeding and molt to occur. We found that these species typically exhibited low breeding period seasonality followed by synchronized peaks in molt intensity and molt‐breeding overlap during peak rainfall and high invertebrate abundance. There was also greater diet overlap and wider niche widths of invertebrate resources in the wet season when molt‐breeding overlap occurred, and greater niche partitioning of invertebrate items among species in the dry season suggesting that competitive interactions for invertebrates were stronger in the dry season. Birds also shared more plant food sources in the dry season when invertebrate abundance is low, though seasonal differences in plant diet diversity and niche width varied by species. These results provide evidence that scarce invertebrate resources and competition likely limit productivity and molt/self‐maintenance in these island‐adapted, species‐depauperate communities, and drive high rates of molt‐breeding overlap, a relatively uncommon life‐history strategy.

## Graphical abstract

Bird populations on small, species depauperate islands often experience selection for generalist foraging traits via ecological release; however, it is unclear how these conditions influence other life‐history characteristics of small‐island birds. We test the explanatory power of three hypotheses to explain the unusually high rates of molt breeding overlap found in an island‐adapted community of birds on the tropical island of Grenada, West Indies. We conclude that competition for scarce invertebrate resources among generalist foragers likely limits productivity and molt/self‐maintenance in these island‐adapted species, and drives high rates of molt‐breeding overlap, a relatively uncommon life‐history strategy.

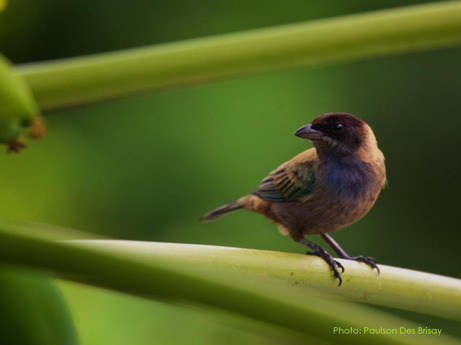

## 1 Introduction

Theory and empirical observation suggest that birds on small, species‐depauperate islands undergo selection for generalist foraging via ecological release, and frequently occupy relatively wide foraging niches and exhibit greater niche overlap compared to species‐rich communities on larger islands or mainland environments (Pianka 1974; Cox and Ricklefs 1977; Terborgh and Faaborg 1980; Wunderle 1985). A broadly recognized pattern in island biogeography is that large and less isolated islands contain greater species diversity than smaller, more isolated islands (Diamond 1974; MacArthur and Wilson 1967; Ricklefs and Lovette 1999; Terborgh, Faaborg, and Brockmann 1978). Here, lower species richness and density compensation drives an increase in intraspecific competition relative to diffuse/interspecific competition in island populations (MacArthur, Diamond, and Karr 1972; Ribon and Marini 2016; Ricklefs and Cox 1978). Cumulatively, these conditions result in selective pressures for adaptations that confer a competitive advantage to individuals that can exploit a wider array of resources or habitats (e.g., Scott et al. 2003). These adaptations could include greater phenotypic plasticity and/or behavioral, morphological, or physiological changes that facilitate broader habitat occupancy and wider foraging niches within or among individuals, a phenomenon commonly referred to as “ecological release” (Bolnick et al. 2003; Costa et al. 2008; Cox and Ricklefs 1977; Van Valen 1973). A further consequence is that species‐depauperate communities also typically exhibit greater niche overlap among species than more diverse communities (Pianka 1974; Bolnick et al. 2003).

In the West Indies, much has been learned about how selection for generalist traits through ecological release from pathogen pressures and interspecific competition influences the colonizing ability (e.g., Arrendt 2006) and habitat distribution of island species (e.g., Vázquez‐Plass and Wunderle 2013; Ricklefs and Cox 1978; Wunderle 1985). For example, theory predicts that Grenadian species have been subject to selection for generalist foraging, which has received support by Wunderle (1985), who reported that bird populations on Grenada occurred in a wider variety of habitats and at higher density than populations on species rich Tobago, and by Heathcote et al. (2021), who found evidence of morphological differences thought to facilitate generalism in some species on Grenada. Similarly, De Ruyck and Koper (2024a, 2024b) also found that Grenadian species typically exploited a wide variety of invertebrate and plant diet items across diverse agricultural and forested landscapes. However, it remains unclear how insular adaptations facilitating generalism may interact with other evolutionary or environmental drivers to uniquely shape the ecology and life‐history characteristics of small island‐adapted birds (Grant 2001; Ricklefs and Bermingham 2008; Wright and Steadman 2012). For example, De Ruyck (2023) also found that Grenadian species exhibited uniquely high rates of molt‐breeding overlap compared to those reported elsewhere for similar tropical species and foraging guilds (e.g., de Araujo et al. 2017; Johnson, Stouffer, and Bierregaard 2012; Silveira and Marini 2012).

### 1.1 Molt Breeding Overlap

Breeding and molt are the two most energetically costly activities undertaken by non‐migratory birds, and are typically separated in the annual cycle due to energetic constraints (Barta et al. 2006; Beltran, Burns, and Breed 2018; Dawson 2008; McNamara and Houston 2008; Piersma 2002; Saino et al. 2014; Wingfield 2008). For example, reduced brood size and growth, reduced feather quality, and reduced immune system function are all costs associated with overlapping breeding and molt (e.g., Barta et al. 2006; de Araujo et al. 2017; Ellis et al. 2012; Johnson, Stouffer, and Bierregaard 2012). However, molt‐breeding overlap occurs to a limited degree in many tropical species, and appears connected to a syndrome of life‐history features favoring adult survival over fecundity that are common to tropical birds such as small clutch sizes, high longevity, low basal metabolic rate, and long molt duration (Barta et al. 2006; Echeverry‐Galvis 2012; Johnson, Stouffer, and Bierregaard 2012; Stutchbury and Morton 2008). Other factors contributing to the viability of molt‐breeding overlap in the tropics include warm temperatures and low seasonal climatic variation that reduces the thermoregulatory costs of poor feather quality incurred by overlap (Beltran, Burns, and Breed 2018; Echeverry‐Galvis and Hau 2013) and the absence of long‐range migration, which reduces time constraints on molt and the survival costs of poor feather quality related to flight efficiency (Echeverry‐Galvis and Hau 2013; Saino et al. 2014). Similarly, lower predator density on islands may reduce the mortality costs of poor‐feather quality. In any case, molt‐breeding overlap is still relatively uncommon among tropical species (but see Echeverry‐Galvis 2012; Johnson, Stouffer, and Bierregaard 2012; and Table 1 displaying rates of molt‐breeding overlap observed in Grenadian birds, De Ruyck 2023).

**TABLE 1:** Rates of molt‐breeding overlap in Neotropical birds reported from previous studies in regions with varying annual rainfall.

Location, # Species/Family, and number of females sampled	Percent of breeding females	Annual rainfall (mm)	Source
Manaus, Brazil, humid mixed forests			
Tyrannidae/flycatchers; 14 sp., *n* = 108	4.4%	2500 mm	Johnson, Stouffer, and Bierregaard (2012)
Thraupidae/tanagers; 4 sp., *n* = 29	0		
Turdidae/thrushes; 1 sp., *n* = 23	0		
Thamnophilids/ant‐shrikes; 21 sp., *n* = 617	23%		
Troglodytidae; 4 sp., *n* = 26	12%		
Vireonidae; 2 sp., *n* = 19	5.6%		
Thirty‐one species with overlap, average % overlap:	12.7%		
Sao Paolo, Brazil, dry deciduous tropical forest			
Ten species with overlap, average % overlap:	15.2%	< 1600 mm	de Araujo et al. (2017)
Costa Rica, dry and humid‐evergreen forests			
Fourty‐seven species with overlap, average % overlap:	8.1%	1500–3300 mm	Foster (1975)
Minas Gerais and Goia State, Brazil, Cerrado mesic and semi‐deciduous forest			
Twenty‐six species with overlap, average % overlap:	8.3%	1500 mm	Marini and Durães (2001)
Estação Ecológica de Águas Emenda‐ das Brazil, Cerrado mesic and semi‐deciduous forest			
Tyrannidae/flycatcher; 1 sp., *n* = 137	4.0%	1500–1750 mm	Silveira and Marini (2012)
Thraupidae/tanager; 2 sp., *n* = 67	12.9%		
Passerellidae/Sparrow; 1 sp., *n* = 14	20.0%		
Five species with overlap, average % overlap:	7.9%		
Grenada, humid mixed forest and agroforests			
Mimidae/mockingbird; 1 sp., *n* = 10	20.0%	1750–2250 mm	De Ruyck (2023)
Tyrannidae/flycatcher; 1 sp., *n* = 6	33.3%		
Thraupidae/tanagers; 4 sp., *n* = 151	34.7%		
Turdidae/thrush; 1 sp., *n* = 27	24.4%		
Troglodytidae/wren; 1 sp., *n* = 10	0.0%		
Vireonidae/vireo; 1 sp., *n* = 6	0.0%		
Nine species with overlap, average % overlap:	21.0%		

*Note:* Reported as % females with an active brood patch undergoing wing molt, *n* = number of females with active brood patch captured in mist‐nets.

### 1.2 Hypotheses and Predictions

The following are three non‐exclusive hypotheses proposed to explain why molt‐breeding overlap is exhibited in tropical species, which we have used to construct predictions to evaluate the explanatory power of each in relation to the uniquely high rates of overlap observed on small island species on Grenada (summarized in Table 2).

**TABLE 2:** Hypotheses and predictions for drivers of molt‐breeding overlap in Grenadian Birds.

Hypothesized driver		H_0_	H_1_
(i)	Long duration molts and temporal constraints induce overlap.	Molt duration correlates with rates of overlap rate across species.	No correlation between molt duration and overlap.
(ii)	Stochastic food availability selects for flexible life‐cycles enabled by overlap.	Peaks in molt‐breeding overlap similar within foraging guilds, but differ across foraging guilds	Peaks in overlap do not vary by foraging guild among species.
(iii)	Short windows of invertebrate availability drive overlap; (iiia) environment limits availability; (iiib) competition significantly limits availability	Peaks in molt‐breeding overlap strongly synchronize with annual rainfall across species.	Peaks in overlap asynchronous with rainfall and/or across species
		(iiia) Species' invertebrate niche widths contract on preferred prey in rainy season and expand to include alternative prey in the dry season, seasonal patterns in diet‐niche overlap vary across species	(iiib) Invertebrate niches expand in wet season to incorporate additional prey and contract in the dry season as species specialize on prey they can best extract, diet niche overlap greater in the wet season than the dry season across species.

Hypothesis (i) long duration molts induce molt‐breeding overlap due to temporal constraints. Johnson, Stouffer, and Bierregaard (2012) found that higher rates of molt breeding overlap correlated with species exhibiting lower feather growth rates and long molt duration. Long‐duration molt may be driven by constraints on molt intensity such as limited protein/invertebrate availability (Echeverry‐Galvis 2012; Harper and Skinner 1998; Murphy and King 1992), high tropical pathogen loads that require greater energetic investment in immune system function (Bailly et al. 2016; Machado‐Filho, Balsamão, and Marini 2010; Møller 1998; Moreno 2004), or flight performance requirements (e.g., hawking insectivores) that limit molt intensity and thus molt rate (Echeverry‐Galvis and Hau 2013). We predicted that if molt duration is a strong driver of molt‐breeding overlap, then species with longer molt duration will exhibit greater overlap.

Hypothesis (ii) unpredictable changes in local food abundance select for flexibility in timing of life‐cycle stage. Life‐cycle theory predicts that combining life‐cycle stages together such as breeding and molt can reduce the temporal constraints on each phase of an individual's annual cycle to enable greater flexibility in responding to temporally unpredictable changes in resources (Barta et al. 2006; Beltran, Burns, and Breed 2018; McNamara and Houston 2008; Wingfield 2008). In tropical forests, fleshy fruit species, an important food source for insects as well as frugivorous birds, exhibit local scale seasonal variability in regards to their flowering, fruiting, and seed dispersal and germination conditions (Ghazoul and Shile 2010; Janzen 1975; Kricher 1997). Thus, local variation in fruit and flower availability are temporally unpredictable in comparison to regionally scaled variation in seasonal rainfall and invertebrate abundance. We predicted that if stochastic food availability drives molt‐breeding overlap, then peak breeding, and molt would be similar among species within foraging guilds, but differ among species in different foraging guilds, assuming fruit, and flower abundance generally varies locally and asynchronously with invertebrate abundance (e.g., Faccio, Gabriel, and Pizo 2018).

Hypothesis (iii) short windows of invertebrate availability force molting and breeding to overlap, (iiia = environmental factors limit food availability independently of competitive interactions; iiib = inter and intraspecific competition significantly contributes to limiting periods of high food availability). There is evidence indicating that seasonally dry regions in the Neotropics with long periods of scarce invertebrate availability can drive higher rates of overlap so that bottom‐up regulated species' populations can exploit the superabundance of resources that are only available over short rainy seasons (de Araujo et al. 2017; Marini and Durães 2001; Payne 1969). In these regions, the short time period over which food is abundant restricts the time available for breeding and molting so that the benefits to reproduction enabled by overlap may outweigh the associated costs. Similarly, gap‐specialists dependent on foods produced within ephemeral tree‐fall habitats, or species in the Thamnophilidae that specialize on following army ants, also exhibit elevated rates of molt‐breeding overlap likely because of the local time constraints on invertebrate abundance imposed by temporary/moving food resources (Johnson, Stouffer, and Bierregaard 2012). We predicted that if limited periods of invertebrate availability drive overlap, then rates of molt‐breeding overlap should strongly synchronize with the predictable four‐fold peaks in invertebrate abundance observed in the mid‐wet season that correspond to peaks in seasonal rainfall (Tanaka and Tanaka 1982).

Hypothesis (iiia) Environmental factors limit invertebrate availability to drive molt breeding overlap. If birds are feeding optimally, molt, and self‐maintenance are not density dependent, and invertebrate availability is primarily a function of invertebrate abundance, then we predict that the community's and individual species resource niche widths should contract in the wet season when diets converge on the highest quality/most abundant diet items, and then niche widths expand in the dry season when high‐quality invertebrates are scarce and individuals seek alternative food sources (Schoener 1974; Stephens and Krebs 1987; Stephens et al. 2019).

Hypothesis (iii‐b) increased intra and interspecific competition for shared invertebrate resources among island‐adapted generalist birds constricts the window of increased invertebrate availability. Grenada does not have a short rainy season similar to dry/semi‐arid regions, and invertebrate biomass has previously been observed to remain elevated with the advent of rains over June to December at an approximately two‐fold increase compared to January–May, with a four‐fold peak in August (Tanaka and Tanaka 1982). However, we hypothesize that competition may be high relative to seasonal increases in invertebrates because of the prevalence of generalist foraging and shared diet items among these species. In addition, increased competition for invertebrates may arise from elevated population densities resulting from generalist foragers increasing their reliance on shared plant food resources in the dry season (De Ruyck and Koper 2024a), which likely elevates survival in agroforests when invertebrates are scarce. We predicted that if periods of high invertebrate availability were limited by competition, then high levels of resource competition would be evidenced by greater niche partitioning (less diet overlap) of scarce invertebrate resources in the dry season among species, while wet season peaks in invertebrate abundance should result in greater niche overlap of high quality invertebrate items in the wet season when competition is relaxed (Bolnick et al. 2010; MacArthur and Pianka 1966; Schoener 1974). Further, competition theory suggests that species should exhibit broader population resource niche widths in the wet season when species expand the range of items consumed to include the wide abundance of high‐quality invertebrates available, while resource niche‐widths contract in the dry season as species specialize on the resource‐types they can best extract (Schoener 1974; Stephens et al. 2019).

To evaluate the explanatory power of these hypotheses and test their predictions we examined data collected on 10 Grenadian species using mist‐netting and banding to estimate the molt duration, phenology, and degree of population synchrony exhibited in breeding, molting, and molt‐breeding overlap for each species. We also used DNA meta‐barcoding of fecal samples to evaluate whether seasonal changes in niche breadth and overlap among species suggest that diffuse competition is stronger in the dry season and limits the time period over which invertebrate abundance is sufficient for breeding and molting.

## 2 Methods

### 2.1 Study Site

The island state of Grenada (312 km^2^) is located in the Caribbean Sea between latitudes 11°59′ and 12°20′ North, and longitude 61°36′ and 61°48′ West. Much of the landscape is elevated and steeply sloped owing to the island's volcanic origins (max. elevation 840 m), and is characterized by a diverse, fine‐grained patchwork (e.g., 1–100 ha) of subsistence and commercial vegetable cropping, agroforestry, and semi‐natural tropical forests, with more forest and agroforestry occurring on slopes and high elevations in the central and northern regions of the island, and more garden and row‐cropping in the southern, eastern, and lowland areas (Helmer et al. 2008; Wunderle 1985). Only 5.9% of Grenada's land area is devoted to arable crops, typically occurring in lowland and plateau belts; 29% of the land is covered by agroforestry production (Helmer et al. 2008), consisting of small mixed stands of cocoa, nutmeg, citrus, breadfruit, cinnamon, avocado, and other fruit (Grenada, 2016); and an additional 50% of the land is forested including dry‐lowland and montane cloud forests, and variously aged secondary forests (Helmer et al. 2008), many of which still contain food trees from the plantation era (*pers obs*).

The dry season is approximately January–May, the wet season is June–December, and the wettest months are July–September. Annual average temperature ranges from 24.2°C (Jan–Feb) to 26.5°C (May). The climate has an extended tropical cyclone season from June to November; however, the last major hurricanes to hit Grenada previous to this research were Janet in 1955, Ivan in 2004, and Emily in 2005. The human population size is ~107,000 (World Bank 2019) and is concentrated along the coastlines in the southwest and east central portions of Grenada, although 70% of the population is considered rural.

Grenada's small size, isolation and heterogeneous landscape has given rise to a relatively depauperate (Appendix S1: Data S1), but densely populated avifaunal community with many species found throughout habitats across the island (Groome 1970; Lack and Lack 1973; Wunderle 1985; Williams, Warrington, and Koper 2023). There are 35 resident land‐bird species on Grenada. Many of the resident land‐birds are subspecies with extremely restricted ranges (Rusk 2003; Rivera‐Milán et al. 2015). Few terrestrial wintering Neotropical or Austral land‐bird migrants are found in most years on Grenada (*pers obs*, Gerbracht and Levesque 2019), limiting the effects of seasonal changes in bird density and interspecific competition. Species commonly captured in terrestrial habitats include: Bananaquit ( *Coereba flaveola* ; nectarivorous/frugiverous), Lesser Antillean Bullfinch ( *Loxigilla noctis* ; frugiverous), Spectacled Thrush (*Turdis nudigenis*; frugiverous), Black‐faced Grassquit ( *Tiaris bicolor* ; graniverous), Lesser Antillean Tanager (*Tangara cucullate*; frugiverous), Grenada House Wren ( *Troglodytes aedon grenadensis* ; insectiverous), Tropical Mockingbird (*Mimus polyglottus*; frugiverous), Black‐whiskered Vireo ( *Vireo altiloquus* ; insectiverous), Grenada Flycatcher ( *Myiarchus nugator* ; insectiverous), and Yellow‐bellied Elaenia ( *Elaenia flavogaster* ; insectivorous; Clements et al. 2021).

### 2.2 Field Methods

**FIGURE 1:**
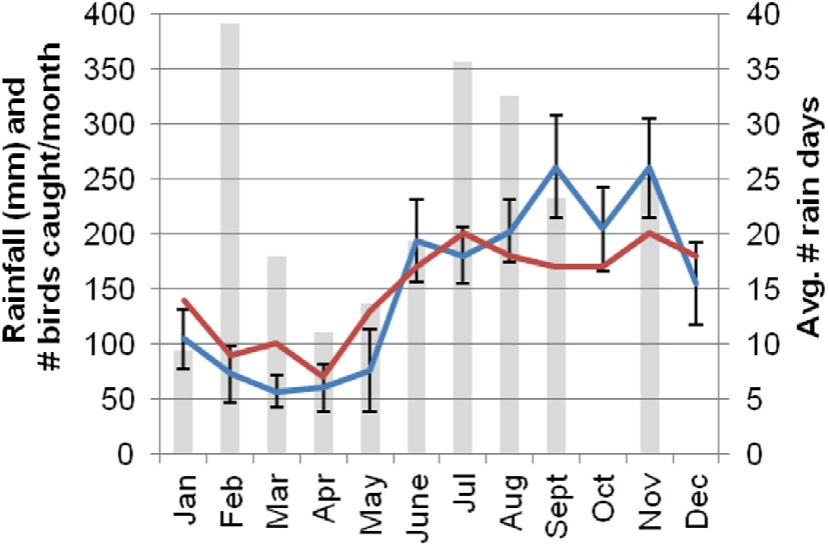
Average monthly rainfall of Grenada's northern interior and 95% confidence intervals (blue line, left axis), recorded within 1.5 km and 100 m elevation of all six capture sites (2009–2018), # birds captured per month (gray bars, left axis), and average # days receiving rain each month (red line, right axis), rainy season begins late‐May to June, and ends December, Grenada (UNEP 2006).

We captured birds at six study sites located within 2.5 km of each other at low to mid‐elevations (75–215 m) in the north of the island over 2015–2019. These sites were a mix of home‐gardens, row‐cropping, mixed‐fruit orchards (e.g., *citrus* spp., *Persea americana* , *Musa* spp., *Mangifera* spp.), mixed‐species forests (semi‐deciduous and evergreen broadleaf), and cocoa‐nutmeg plantations ( *Theobroma cacao* and *Myristica fragrans* ), located at Belmont Estate, 12.176  N 61.627  W, and Coubarri, 12.163  N 61.643  W. All sites were organically farmed without pesticides. We captured birds in mist‐nets, measured biometric characters, and classified breeding condition, molt, age, and sex according to methods described by De Ruyck et al. (2024a), adopted from Redfern and Clark (2001), and Wolfe, Ryder, and Pyle (2010). We collected feces in sterile Eppendorf tubes from cloth bags used to hold captured birds as described by De Ruyck and Koper (2024a), and collected fecal samples opportunistically in the wet season June 1, 2018 to September 13, 2018, and over the dry season May 2–20, 2018, and January 29, 2019–March 31, 2019 (Figure 1).

### 2.3 Laboratory Methods

#### 2.3.1 DNA Extraction, Amplification, and Sequencing

We used Qiagen Fast DNA Stool Mini kits to extract DNA from samples collected in 2018, and Qiagen DNeasy Powersoil kits to extract DNA from samples collected in 2019 and some additional samples from 2018. We changed kits to see if we could improve the purity of DNA extracted from Spectacled Thrush samples, as PCR inhibitors appeared to limit successful amplification of samples obtained from larger birds with the stool kits. In all cases, we extracted DNA from the entire fecal sample (dry weight < 50 mg), and modified the manufacturer's recommended protocols according to methods described by Trevelline et al. (2016) and Zeale et al. (2011) to improve DNA yield and quality. We submitted samples as DNA extracts to the Canadian Centre for DNA Barcoding (CCDB) using their dry storage preservation microplates. De Ruyck and Koper (2024a) provides full descriptions of the DNA extraction, PCR, sequencing, and bioinformatic methods used for this study. We report data from all species in which we collected 10 or more fecal samples.

### 2.4 Statistical Analysis

#### 2.4.1 Molt‐Breeding Overlap

To make a conservative estimate of the amount of molt‐breeding overlap, we restricted our analysis to adult birds, and classed males in breeding condition if they scored 2 or 3 on a scale of 0–3 based on the enlargement of their cloacal protuberance, and we classed females in breeding condition if they scored 2–4 on a scale of 0–5 based on the appearance of their brood patch (Redfern and Clark 2001). We classified birds in active wing‐molt when we observed adults symmetrically replacing two or more feathers on a wing in sequence. We did not exclude individuals undergoing wing molt without tail molt as recommended by Johnson, Stouffer, and Bierregaard (2012) as we observed tail molt to be variable and often asymmetric or adventitious in members of the Tanager family, and thus an unreliable indicator of the definitive molt cycle. We assessed bird age based on an examination of molt pattern, plumage, and feather quality (wear, shape, and barbule density), and in conjunction with skull ossification when necessary/appropriate (Redfern and Clark 2001). We examined the degree of molt‐breeding overlap exhibited by each species by calculating the percentage of total adult birds captured each month that exhibited active wing‐feather molt while in breeding condition. We also calculated the percentage of the total number of breeding adult females captured (all months combined) that exhibited wing‐feather molt while in breeding condition as reported by other researchers (e.g., Johnson, Stouffer, and Bierregaard 2012; de Araujo et al. 2017).

#### 2.4.2 Molt Duration and Synchrony

We used Pimm's regressions (Pimm 1976) to estimate the molt duration of Grenadian birds as described by De Ruyck (2023). We examined the degree to which molt duration may influence the rate of molt‐breeding overlap using linear regression with the calculated percentage of molt‐breeding overlap as the dependent variable, and molt duration estimates as the independent variable for each species for which we had sufficient sample size to calculate a molt duration estimate (*n* = 8 species). We also calculated the intensity of molt (number of flight feathers concurrently molting) averaged for each species per month to visualize molt synchrony within and across species.

#### 2.4.3 Diet Analysis

We analyzed diet items at the level of genus to conservatively avoid potential problems with mis‐identifications at the species level (Pompanon et al. 2012). To examine the degree of niche overlap among species and between years, we calculated resource utilization matrices by compiling counts of occurrences of unique operational taxonomic units (at the genus level) to calculate frequency of occurrence values for each diet item by dividing the number of samples in which a diet genus was identified by the total number of samples analyzed (per species). Frequency of occurrence data may overestimate the importance of items consumed in small quantities, but better representativeness is obtained when the average number of prey items present per sample is small (Deagle et al. 2019; Pompanon et al. 2012), such as is the case with small passerines with relatively fast metabolism and small stomachs. Frequency of occurrence resource matrices were calculated for each bird species, for each season (wet or dry), and we also separated plant and invertebrate items to calculate the frequency of occurrence of insects and plants separately for each species per season.

To quantify the degree of niche overlap among species, we used the “niche.overlap” function from the “spaa” R package (Zhang 2016) to calculate Pianka's niche overlap index values for plants and insects separately for each species pair for each season (Pianka 1973). We also calculated the average community niche overlap index for each season for insects and plant items. We dropped Spectacled Thrush from these analyses because of insufficient sample size. We also repeated these calculations based on the occurrences of unique orders rather than genera. In addition, we repeated these calculations after removing diet items that only occurred once among all samples for each species. This procedure reduced the total number of diet items by approximately 20%; however, it restricts the analysis of diet to items that we can be more confident are regularly included and energetically important in the diet of each species (Pompanon et al. 2012). For simplicity, we present figures based on the full dataset unless otherwise noted; analyses using the truncated data set of items consumed more than once or based on frequency of occurrence at the level of order rather than genus all yielded similar results. We used ANOVA and Tukey pair‐wise comparisons of estimated marginal means (Quinn and Keough 2006) to test for differences in overlap indices between seasons among species pairs for invertebrate and plant items separately. Here, the Pianka overlap index for each species pair was the dependent variable, and species pair, and season (wet and dry) were the explanatory variables.

To assess whether sample sizes were sufficient to adequately describe the diet of this bird community, and compare diet diversity across seasons, we used the “specaccum” function of the vegan R package (Oksanen et al. 2020) to calculate rarefaction curves using the number of unique genera identified in each fecal sample per species for each season, and randomized samples 999 times. As a separate analysis of diet diversity and resource‐breadth, we calculated Levin's niche‐breadth (MacArthur and Levins 1967) and Shannon's diversity indices separately for invertebrate and plant items for each species using the spaa R Package (Zhang 2016). We then used ANOVA and Tukey pair‐wise comparisons of estimated marginal means (Quinn and Keough 2006) to test for differences in niche breadth and diversity between seasons averaged among species for insects and plant items separately. Here, Levin's niche breadth or Shannon's diversity was the dependent variable, and species, and season (wet and dry) were the explanatory variables. We evaluated the fit of all models and assumptions of homoscedasticity using diagnostic plots of residuals and standardized residuals versus fitted values, residuals versus factor levels, and Q‐Q plots.

In all cases, results from our diet meta‐barcoding data must be considered cautiously due to these caveats: (1) fecal samples collected over wet and dry seasons spanned approximately 3 months each, and thus frequency of occurrence counts are not a “snapshot” of diet conditions at any one time, but are aggregates of foraging behavior spread over longer time periods, which potentially can over‐or under‐estimate the amount of diet overlap among species; (2) small stomach sizes may constrain the number of diet items recorded per individual, and therefore, underestimate the diet breadth of individuals, but overestimate variation within a population or overestimate variation between species (Pompanon et al. 2012); and (3) variability in successful amplification or sequencing of different diet items may also skew the proportions of diet genera represented in the data (e.g., Deagle et al. 2019; Neby et al. 2021).

## 3 Results

### 3.1 Generalist Foraging Niches

We analyzed 344 fecal samples from 10 bird species and identified plant and/or invertebrate DNA from 84% of samples, ranging from 59% of Spectacled Thrush samples to over 90% of samples for seven other species (Table 3). All species frequently consumed invertebrates in the wet and dry seasons including birds commonly thought to be chiefly frugivorous or nectarivorous (Table 3, Appendix S2: Data S1), consistent with predictions of diet generalist foraging in small‐island populations. We did not test for vertebrate DNA, though we observed Grenada Flycatchers and Tropical Mockingbirds eating *Anolis* lizards. There were 190 invertebrate genera identified, and 66 plant genera identified. We also found that the most commonly occurring invertebrate and plant genera were consumed by many bird species, for example, *Raphanus* (shared by eight bird species), *Paspalum* (seven species), *Clidemia* (seven species), *Psidium* (eight species), and *Andira* (seven species) were the most commonly occurring plant genera; and *Argiope* (shared by seven species), *Drosphila* (six species), *Antillattus* (six species), an unidentified genus of Cecidomyidae (six species), and an unidentified Lepidoptera (six species) were the most commonly occurring invertebrate genera.

**TABLE 3:** The number of samples per bird species with identifiable invertebrate and plant DNA, Grenada, 2018–2019.

Common name	Local name	% with invertebrates	% with plants	*n* samples with DNA	*n* total samples
Bananaquit	See‐see bird	89%	87%	63	64
Lesser Antillean Bullfinch^a^	Red‐throated See‐see	60%	92%	64	70
Lesser Antillean Tanager^a^	Sour See, or Soursop Bird	56%	100%	31	43
Black‐faced Grassquit	See‐see, or Grass See‐see	39%	100%	28	33
Spectacled Thrush	Cocoa Grief	47%	92%	42	71
Tropical Mockingbird	Pecao	83%	50%	9	10
Grenada Flycatcher^a^	—	92%	100%	15	15
Yellow‐bellied Elaenia	Top‐knot	33%	89%	12	12
Black‐whiskered Vireo	—	88%	94%	15	15
Grenada House Wren	Guard Bird or House Bird	71%	71%	11	11

^a^ Endemic to the Lesser Antilles.

### 3.2 Hypothesis (i): Molt Duration Drives Molt Breeding Overlap

**FIGURE 2:**
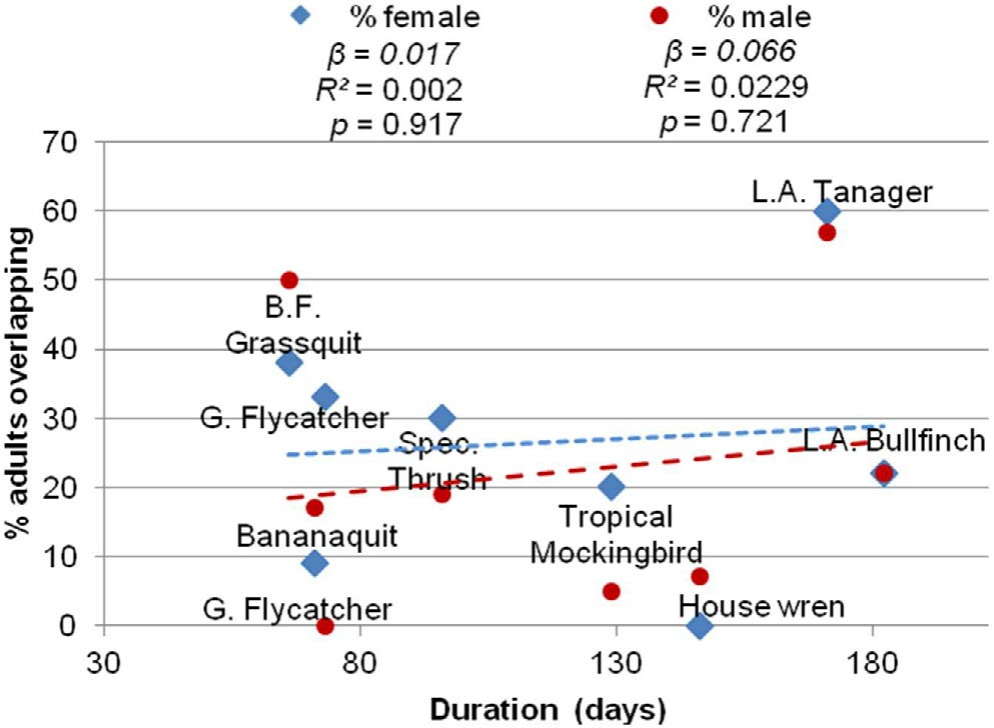
Scatterplot depicting molt duration (x‐axis) against % of breeding adults exhibiting molt‐breeding overlap for females (blue) and males (red), Grenada 2015–2019. We detected no significant relationship among these eight species for males or females.

**FIGURE 3:**
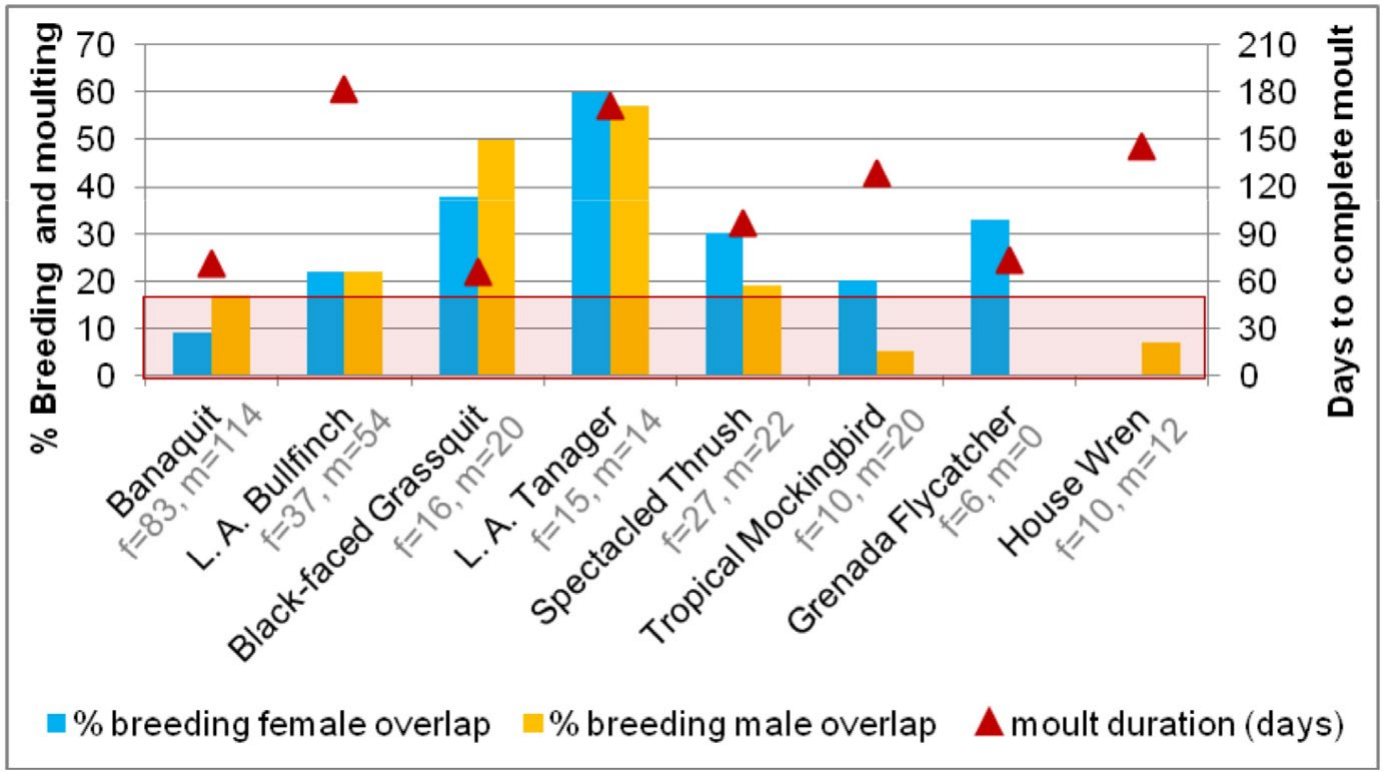
High percentage of breeding adult females and males exhibiting molt‐breeding overlap (blue and yellow bars, respectively) unrelated to estimated molt duration, indicated by red triangles in days on the right axis (calculated from Pimm's regressions except bullfinch and grassquit whose molt duration was calculated from recaptures within the same molt cycle). Shaded red region indicates the average amounts of molt‐overlap reported in other tropical regions.

There is large variation in molt duration among species (average 121 days, ranging from 66 to 182 days; De Ruyck 2023), but we did not detect a significant relationship between the percentage of females exhibiting molt‐breeding overlap and the estimated molt duration exhibited by each species *β* = 0.017, *p* = 0.917, *R* ^2^ < 0.01, *df* = 8, (males: *β* = 0.66, *p* = 0.721, *R* ^2^ = 0.02, *df* = 8; Figure 2), suggesting that long molt duration was not a primary driver of the molt‐breeding overlap observed. For example, the rate of molt‐breeding overlap exhibited by Black‐faced Grassquits and Lesser Antillean Tanagers was much higher than overlap amounts reported for species in other tropical regions; however, the estimated time to complete molt for grassquits was a short 66 days, while the estimated molt duration for tanagers was three times as long at 171 days (Figure 3). Similarly, House Wrens had a long 146‐day molt duration compared to Bananaquits at 71 days, but both species exhibited considerably lower amounts of overlap that are comparable to species/populations elsewhere (Figure 3).

### 3.3 Hypothesis (ii): Stochastic Resource Abundance Drives Molt‐Breeding Overlap

**FIGURE 4:**
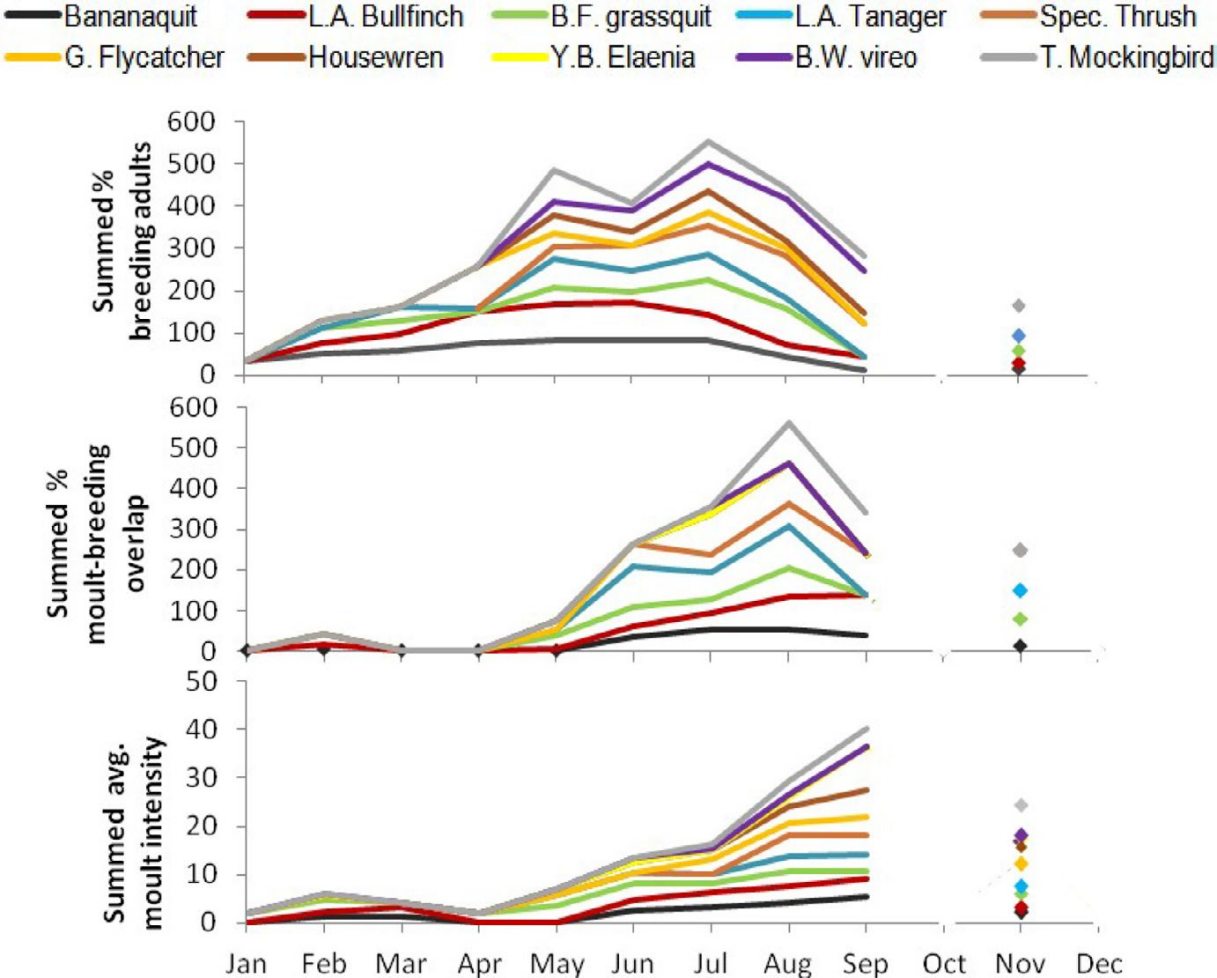
Summed monthly population averages demonstrating common patterns of: Percent of adult birds breeding (top), percent overlapping breeding and molt (middle), and average wing molt intensity (# wing‐feathers simultaneously molting; bottom) across 10 species. Summed percentage can range from 0% = no individuals of any species to 1000% = all individuals of all 10 species). No birds were captured October or December, Grenada, 2015–2019.

The breeding period was protracted with low proportions of the population breeding over the dry season for most species (De Ruyck 2023), but peak breeding generally occurred in the late‐dry season (May) or early rainy season (June–July) across species (top panel, Figure 4). Wing molt was generally absent during the dry season except for low‐intensity molts exhibited in the Thraupidae (bananaquit, grassquit, bullfinch, and tanager). Other species began a low‐intensity molt in the early‐to‐mid rainy season, and all species exhibited a synchronized peak in molt intensity in the mid‐wet season in August–September (bottom panel, Figure 4). Molt‐breeding overlap was rare during the dry season, but we detected low rates in February in bananaquits, bullfinches, and grassquits whose breeding seasons included the early dry season (middle panel, Figure 4). The percentage of breeding adults showing overlap began to rise in the early rainy season (June), and typically peaked synchronously across species in August, and then began to decline from September onwards. These results suggest that the timing of wing molt and molt‐breeding overlap were largely synchronized with predictable peaks in rainfall and invertebrate abundance in August–September (Figures 1 and 4; Tanaka and Tanaka 1982) across species/foraging guilds, and therefore molt‐breeding overlap is unlikely to result from stochastic seasonal variation in food availability.

### 3.4 Hypothesis (iii): Short Windows of Invertebrate Availability Drives Molt‐Breeding Overlap

**FIGURE 5:**
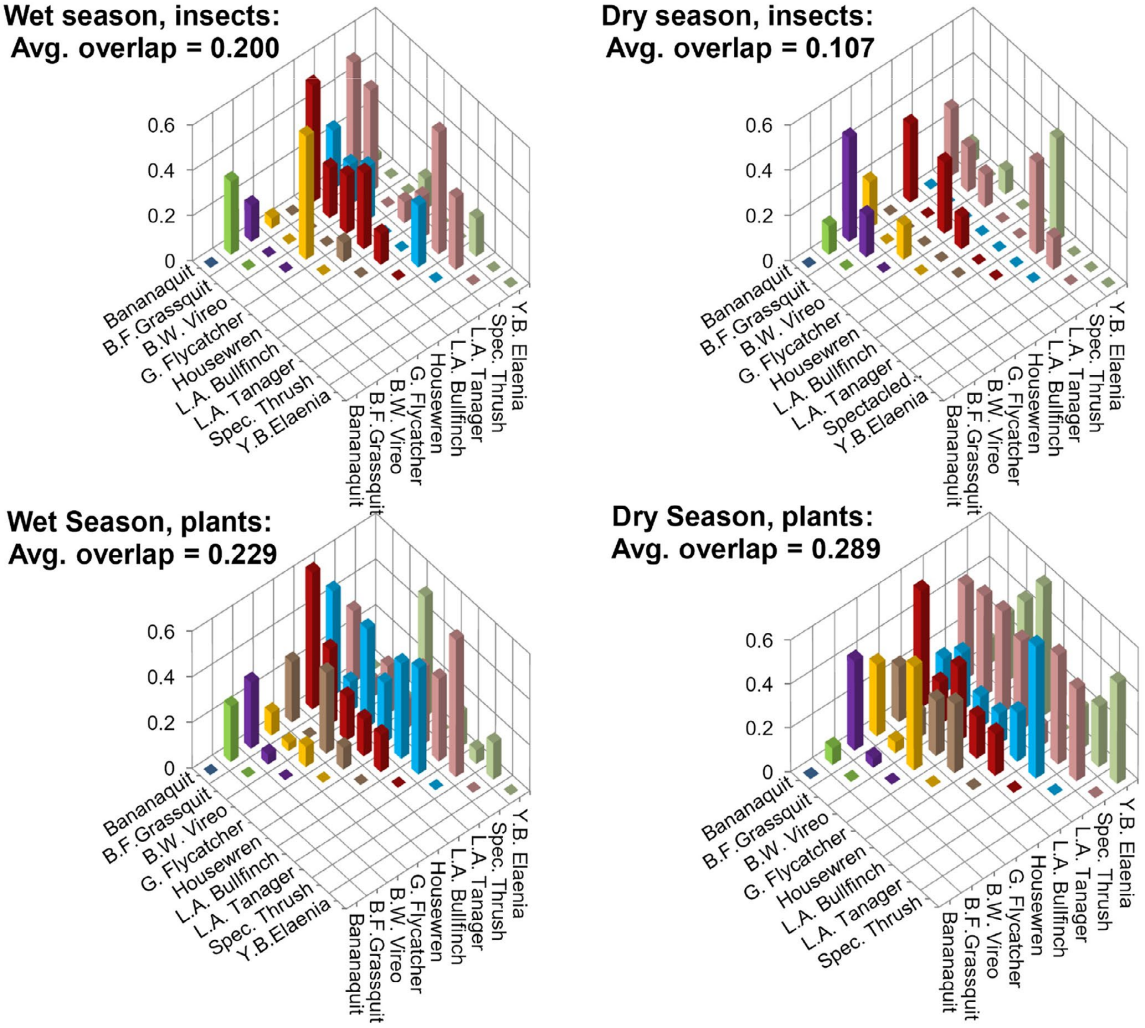
Pianka indices of niche overlap based on frequency of occurrence of diet genera collected over six sites, calculated pairwise across a nine species community for both wet and dry seasons (bird species separated by color along the right hand axis). Overlap of invertebrates consumed higher in the wet season, while overlap of plant items consumed higher in the dry season, Grenada 2018–2019.

**FIGURE 6:**
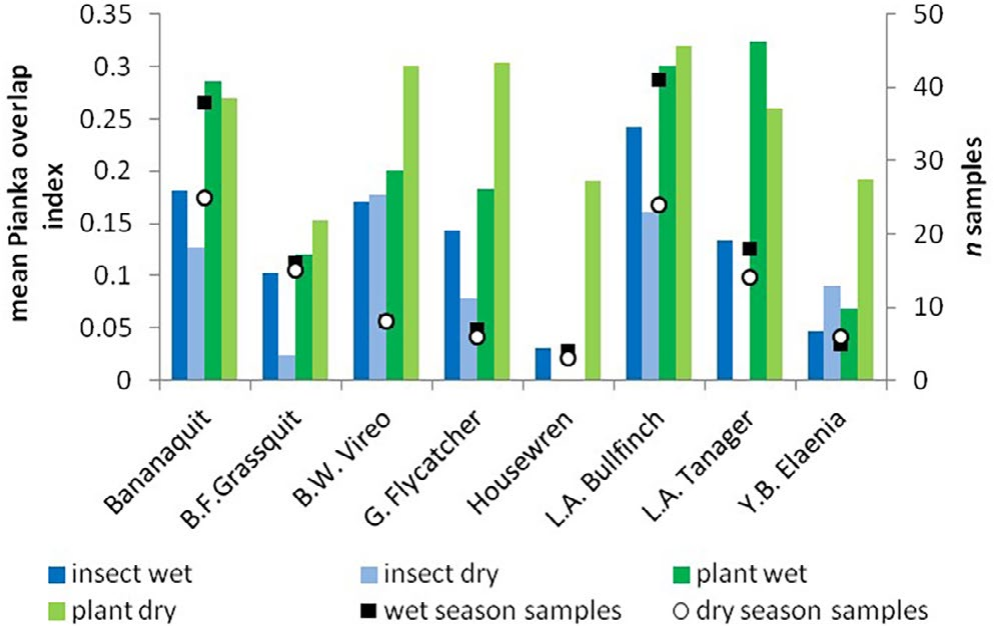
Pianka resource‐niche overlap indices averaged across species pairs for each species per season indicate greater overlap of invertebrate food items in wet season for most species (blue columns), while seasonal patterns of overlap for plant genera were greater in the dry season (green columns). Number of samples per bird species yielding identifiable reads of invertebrate genera (black squares), and plant genera (white circles), Grenada 2018–2019.

**FIGURE 7:**
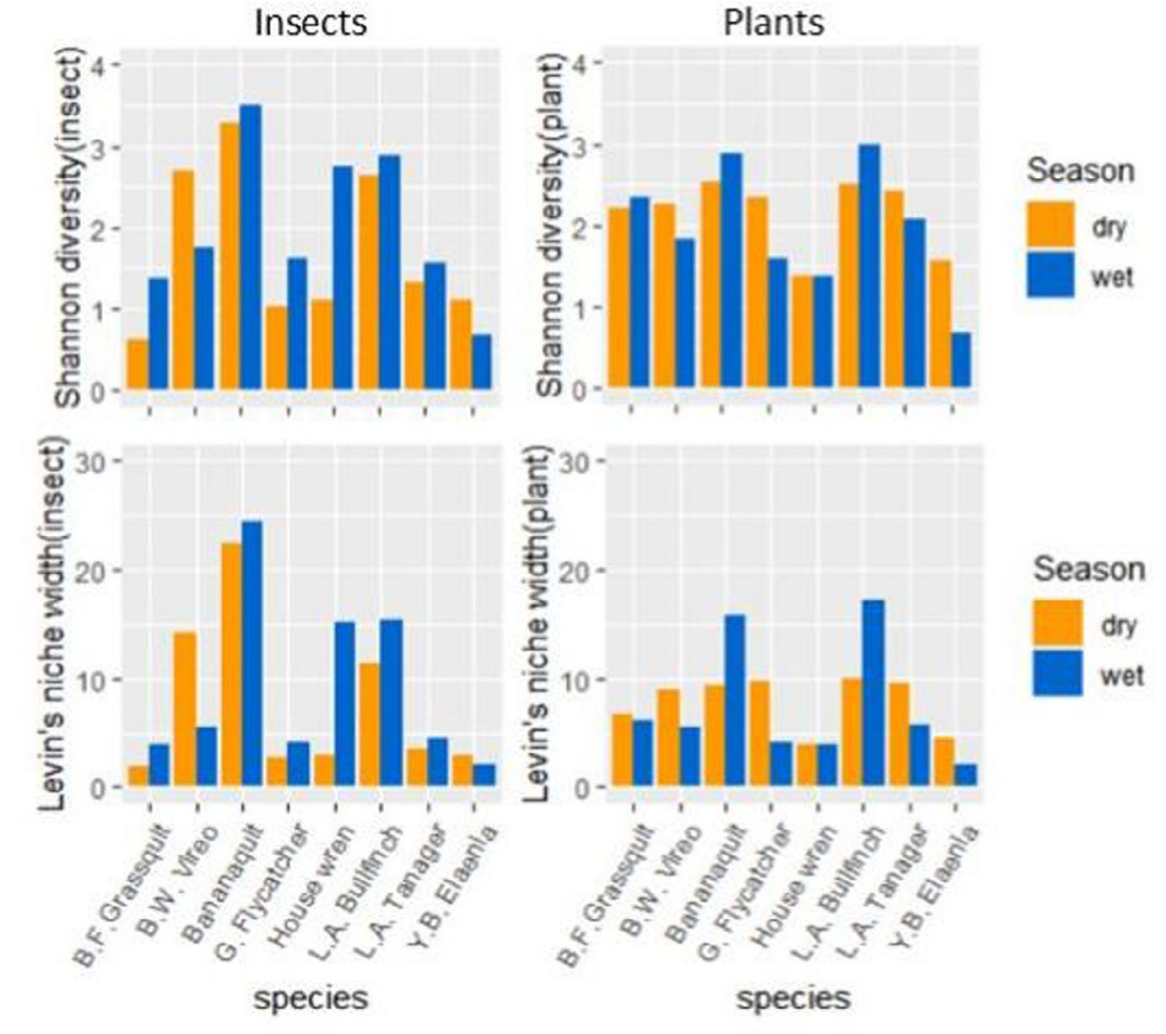
Shannon diversity and Levin's resource niche widths of invertebrates (left panels) are higher in wet season for all species except vireos and elaenias, while seasonal differences in diet diversity and niche width of plant items (right panels) do not show a consistent pattern across species Grenada, 2018–2019.

We observed greater overlap of invertebrate diet resources, and wider invertebrate diet‐widths in the wet season compared to the dry season, consistent with predictions if invertebrate abundance is limited over the dry season. The Pianka niche overlap index of invertebrate diet items was 87% higher in the wet season (0.200) than the dry season (0.107; top panels, Figure 5). In contrast, the amount of niche overlap among plant items was 21% lower in the wet season (0.229) than the dry season (0.289; bottom panels, Figure 5). Similar results were obtained from performing these calculations at the level of order rather than genus (e.g., wet season invertebrate order overlap index = 0.621, dry season = 0.380; wet season plant overlap index = 0.435, dry season = 0.553). Mockingbirds were left out of these calculations due to insufficient sample sizes. Similarly, the overlap indices of invertebrate resources averaged across species pairs were significantly higher in the wet season than the dry season for the truncated data set: *β* = 0.10, *df* = 20, *t*‐ratio = 3.121, *p* = 0.0054 (Figure 6) as the competition hypothesis (iiib) predicted, while plant overlap indices did not significantly vary between seasons: *β* = −0.047, *df* = 27, *t*‐ratio = −1.364, *p* = 0.1837 (Figure 6). Results from the full data set of diet items showed similar trends to the results from the truncated data, but were insignificant. Spectacled Thrushes and Tropical Mockingbirds were left out of these calculations due to uneven and small sample sizes between seasons, respectively. Our results also suggest that species consumed a greater range of invertebrate resources in the wet season than the dry season. Shannon diversity, and Levin's niche width values of invertebrate diet items were higher in the wet season as predicted for every species except vireos and elaenias (Figure 7); however, the estimated marginal means of invertebrate and plant niche widths averaged across all species in the community as a whole were not significantly different between seasons in contrast to predictions (Table 4). This null result for the community is due to the contrasting results from vireos and elaenias compared to the majority of species (Figure 7).

**TABLE 4:** Seasonal comparison of diet diversity and niche‐width using estimated marginal means averaged across all bird species.

	Estimate	*df*	*t*‐ratio	*P*‐value
Shannon diversity				
Invertebrates	0.291	7	1.068	0.3210
Plants	−0.181	7	−1.040	0.3328
Levin's niche‐width				
Invertebrates	1.62	7	0.791	0.4547
Plants	−0.319	7	−0.193	0.8527

*Note:* Positive values signify more diverse and wider invertebrate resource niches in the wet season, but all results non‐significant, Grenada 2018–2019.

**FIGURE 8:**
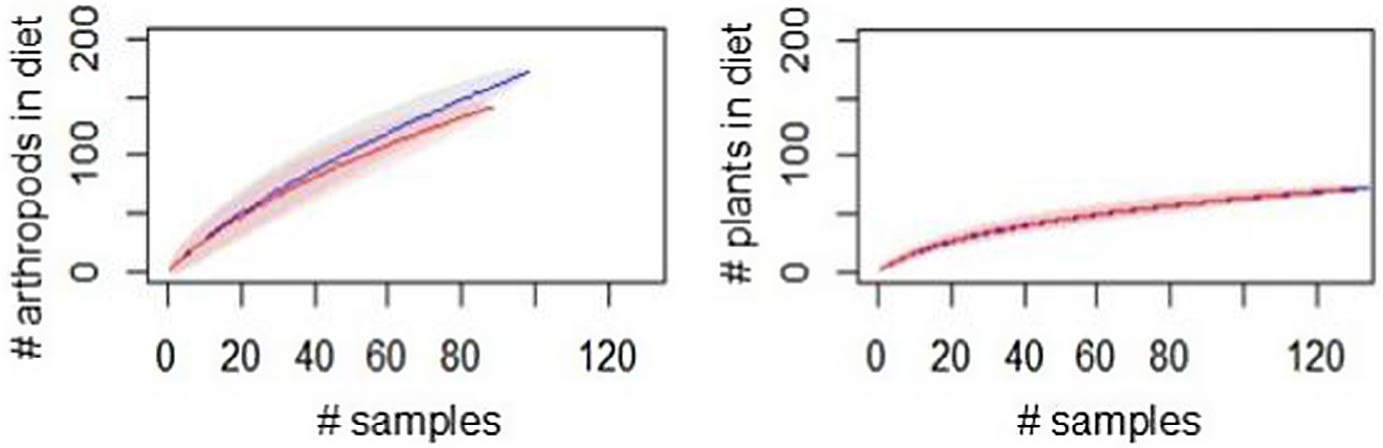
Rarefaction curve depicting accumulation of diet genera occurring in the diet of each bird species (*n* = 9) across six sites with 95% confidence intervals, wet season in blue, dry season in red. Invertebrate genera on left, plant genera on right, Grenada, 2018–2019.

We also observed greater variability among species in the range of invertebrates consumed than variability in plants. For example, Levin's resource niche widths varied significantly across species for invertebrates, but not plants (insects, *df* = 7, *F*‐value = 6.188, *p* = 0.014; plants, *df* = 7, *F*‐value = 2.435, *p* = 0.132). However, the total richness of invertebrate and plant items consumed by the community were not statistically different between seasons (Figure 8), though the lack of a defined asymptote on the curve describing accumulation of invertebrate diet items suggests that the true diversity of invertebrate items is greater than what is represented in our sample. Similarly, examining species accumulation curves for each bird species separately indicated that low sample sizes resulted in inadequate accumulation plateaus that limited our ability to estimate true diet richness at the genus level for these species individually (Appendix S3: Data S1), even as accumulation curves at the level of order had more pronounced plateaus and suggested similar seasonal patterns to those apparent at the level of genera.

Together, the results indicate that though the diversity of invertebrates and plants consumed by this community are relatively similar between seasons, there is more niche overlap of invertebrate prey among species in the wet season when invertebrate abundance is high, for example, *Drosophila* (fruit flies), *Salticidae* (jumping spiders), *Anastatus* (parasitic wasps), and various *Lepidoptera* (butterflies). Further, there is an increased overlap of plant resources consumed in the dry season. This increase is driven in part by insectivores incorporating more plants species in their diet, which largely consist of agricultural plants or plants associated with agricultural/human habitats. Overall, we concluded that hypothesis (iii) is broadly supported and there is some supporting evidence that competition contributes to further limiting the availability of invertebrates over the dry season (iiib).

## 4 Discussion

Our results indicate that seasonal variation in invertebrate abundance plays a definitive role in similarly shaping the life‐cycle phenology of Grenadian species despite differences in their morphology, phylogeny, or presumed foraging guild. These similar patterns in life‐cycle timing and molt‐breeding overlap across species likely result from the prevalence of generalist foraging behavior demonstrated by the wide array of invertebrate and plant diet items shared across bird species. In addition, our analysis provided a means to evaluate the hypotheses posed to explain the high rates of molt‐breeding overlap observed in Grenadian birds, and demonstrates that overlap likely results from the interacting factors of ecological release and generalist foraging, seasonal variation in resource abundance, and inter and intra‐specific competition for invertebrates in these small‐island adapted birds.

### 4.1 Hypothesis (i) Molt Duration

We did not find a significant relationship between molt duration and molt‐breeding overlap. In addition, both fast and slow‐molting species synchronized periods of molt‐breeding overlap with peaks of high invertebrate availability in the mid rainy season, which means that it is unlikely that constraints on molt duration drive high rates of overlap. However, it is worth noting that theory predicts that island species face lower pathogen and predation pressures than mainland species via ecological release (Bailly et al. 2016; Ricklefs and Bermingham 2002), and therefore lower immune system requirements and fewer predators for island birds may also reduce the costs of molt‐breeding overlap (Martin II, Hasselquist, and Wikelski 2006; Moreno 2004).

### 4.2 Hypothesis (ii) Stochastic Variation in Resources

The relatively high rate of synchronicity in molt intensity and molt‐breeding across species and foraging guilds with predictable seasonal rainfall makes it less likely that molt‐breeding overlap is driven by stochastic fluctuations in local food abundance. However, further research on the variability in overlap rates between years is needed to further examine the potential role of resource stochasticity in driving selection for flexible life‐cycle stages on Grenada. For example, Echeverry‐Galvis (2012) found significantly greater rates of molt‐breeding overlap in wet years compared to dry years in dry mountain‐top habitats, even as birds molted consistently both years in the mid to late wet season. Thus, general flexibility in molt intensity and the timing of the breeding period facilitated by overlap may be an important evolutionary adaptation that enables birds to better match life‐cycle phenology to a variety of seasonal resource patterns to increase reproductive rates and enable individuals to offset the energetic costs of poor flight efficiency associated with not molting. This strategy remains consistent with research in other tropical regions demonstrating high population level synchronization of molt to rainfall/invertebrate abundance, while breeding often shows weaker seasonality among individuals or locales (Cox et al. 2013; de Araujo et al. 2017; Faccio, Gabriel, and Pizo 2018; Nwaogu, Tieleman, and Cresswell 2019; Snow and Snow 1964).

### 4.3 Hypothesis (iii) Molt‐Breeding Overlap Enables Species to Benefit Both Reproductively and for Self‐Maintenance via Molt From Short Periods of Invertebrate Protein Sources

The observed patterns in life‐cycle phenology and seasonal differences in diet diversity and niche partitioning suggest that invertebrate scarcity drives overlap in Grenadian generalist‐adapted species (*hypotheses iii a and b*). The across‐species synchronicity of molt‐breeding overlap and molt intensity with peaks in rainfall and invertebrate abundance in August (Tanaka and Tanaka 1982) suggests that peak seasonal availability of invertebrates influences the life‐cycle phenology of these species similarly. In addition, higher Pianka niche overlap indices in the wet season suggest that species' invertebrate diets converge on commonly available diet items when invertebrate abundance is high, consistent with *hypothesis (iii)*. However, there is no data on the relative abundance of different invertebrate prey items with which to assess bird's diet “selectivity;” and the lack of plateaus in the accumulation curves of invertebrate‐diet genera indicate that this dataset provides an incomplete description of the diets of these species. Thus, it is difficult to separate the influence that intra and interspecific competition may have on invertebrate abundance apart from the effects of seasonal variation in abundance because it is unknown to what extent birds are feeding optimally versus opportunistically in either season.

### 4.4 Hypothesis (iii‐a) or (iii‐b)?

If short periods of high invertebrate abundance limited availability independently of competition (e.g., due to a short rainy season; *hypothesis iii‐a*), then theory predicts that optimally foraging species populations should exhibit narrower and less diverse invertebrate diet‐niche widths in the wet season when diets converge on abundant high‐quality invertebrate resources. Likewise, populations should exhibit wider invertebrate diet‐niches in the dry season when individuals seek a diversity of alternative food sources (Schoener 1974; Stephens and Krebs 1987; Stephens et al. 2019). Our results suggested the opposite pattern—consistent with competition theory (*hypothesis iii‐b*)—showing most species exhibiting broader invertebrate diet‐niches in the wet season to incorporate additional commonly available invertebrates when food abundance is high and competition is relaxed, and narrower diet‐niches in the dry season when competition may restrict species to focus on resources that they can best extract and/or exclude other species from (Stephens et al. 2019). However, this same pattern may arise if birds are simply feeding opportunistically (not optimally) on a greater diversity and abundance of prey in the wet season compared to the dry season independently of competition or prey quality. Without data on the abundance and/or quality of prey items relative to their consumption by each species, we cannot confirm the role of competition in limiting the period of invertebrate availability (Sherry 1990; Sih and Christensen 2001). Intriguingly, vireos and elaenias did not exhibit a wider wet season invertebrate diet‐niche as observed in the other species. If competition were playing a role in determining diet‐niche widths, this may have occurred in part because vireos were the only species to frequently occur in the upper canopy of closed‐canopy forests, and therefore potentially experience less impacts from interspecific competition compared to the other species that co‐occurred more frequently across habitats, while the elaenia invertebrate diet data were the most severely restricted by sample size of the species considered, and potentially the least representative of that population's diet.

The seasonal patterns of diet‐niche overlap observed are similarly inconclusive about the influence of competition on limiting invertebrate abundance. Consistent with competition theory, invertebrate diet‐niches overlapped more across species in the wet season when competition is relaxed and species' diets converge on abundant/high‐quality diet items, which provides evidence of greater niche partitioning among species in the dry season when invertebrates are scarce and competitive interactions are stronger (Schoener 1974; Stephens and Krebs 1987). However, this pattern could still arise from opportunistic feeding independently of competition effects if the distribution of mobile invertebrate resources throughout the environment is more sporadic/patchy during the dry season relative to the wet season (Sih and Christensen 2001). For example, specific Lepidoptera and Diptera genera may be ubiquitous and abundant throughout multiple habitat types or micro‐habitats in the wet season, while dry season invertebrate taxa are more limited in their distribution, and therefore differentially encountered by bird species to a greater degree due to species‐specific foraging behaviors or micro‐habitat preferences.

Overall, we conclude that these small‐island adapted species experience an “invertebrate desert” similar to that described by Sherry et al. (2020) to explain the large degree of predator–prey specialization in tropical insectivores arising from intense diffuse competition for invertebrates. In our case, the desert conditions temporarily arise because of seasonal variation in rainfall and invertebrate abundance, which may be further restricted by competition among high densities of generalists. Here, the diversity of plant food resources available throughout the mosaic landscape of garden, agroforest, and secondary forest patches (Bergen et al. 2023; Williams, Warrington, and Koper 2023, De Ruyck and Koper 2024a, 2024b) contributes to supporting adult survival and high population densities, which likely further limits the period in which invertebrate abundance is sufficient for successful breeding and molt to occur to the August–September peak in rainfall (Ashmole 1963). Moreover, competition for invertebrates with high densities of similarly generalist‐adapted *Anolis* lizards present on small islands in the Lesser Antilles may also play a role in limiting periods of invertebrate availability for birds (Wright 1979, 1981; Simmons et al. 2005). We also frequently observed large, mixed flocks of birds congregating on aseasonally blooming and fruiting trees such as the French Cashew (*Syzygium*), Coral Tree/Flame‐of‐the‐forest (*Erythrina*), African Tulip (*Spathodea*), or Strangler Fig (*Ficus* spp. De Ruyck and Koper 2024a, *pers obs*), further suggesting that limited food resources drive non‐territory holding birds to seek spatially variable fruit, nectar, and seed resources (and invertebrates attracted to these trees) for survival, which help maintain bird population densities at elevated levels relative to the availability of invertebrate prey required for reproduction and molt.

Under these small‐island conditions, we speculate that the costs incurred by molt‐breeding overlap such as reduced immune system function, feather quality, or clutch‐size/egg mass (Moreno 2004; Barta et al. 2006; Echeverry‐Galvis and Hau 2013) are outweighed by the benefits of molting and reproducing when invertebrate abundance is high relative to bird population density. These conditions and trade‐offs are similar to other bird populations that exhibit molt‐breeding overlap and face limited time periods of invertebrate abundance, whether these invertebrate desert conditions are induced by diffuse competition (e.g., lowland, humid tropical forest insectivores; Johnson, Stouffer, and Bierregaard 2012; Sherry et al. 2020), the ecology of preferred prey or habitats (e.g., ant followers, and gap specialists; Johnson, Stouffer, and Bierregaard 2012), or climate (e.g., seasonal rainfall in arid/semi‐arid habitats; Silveira and Marini 2012; de Araujo et al. 2017). The high rates of molt‐breeding overlap found across a variety of species/foraging guilds in elevated Colombian mountain tops (Echeverry‐Galvis 2012) also share some common biogeographic features with Grenada's small‐island populations, such as seasonal rainfall, relative isolation, reduced species richness, (Das 1977; Patterson et al. 1998; Kattan and Franco 2004; Herzog, Kessler, and Bach 2005), and potential for ecological release from competitors and/or pathogens. Therefore, similar mechanisms may be driving overlap in these two study systems.

In conclusion, the present study combined with other research suggests that the capacity to overlap breeding and molt in tropical ecosystems enables individuals to flexibly alter the duration of the breeding period to optimize annual reproductive output, while retaining the capacity to synchronize molt/self‐maintenance with predictable peaks in seasonal rainfall and invertebrate abundance. Overlap of breeding and molt may be evolutionary advantageous under several non‐exclusionary conditions such as limited temporal windows of resource abundance, as well as stochastic variability in food abundance among morphologically and phylogenetically diverse species. These conditions of seasonally limited invertebrate availability may commonly arise on tropical small islands and other highly productive ecosystems with similar biogeographic characteristics, seasonal rainfall, and relatively depauperate and generalist‐adapted bird communities. However, much remains to be understood about the strength of competitive interactions among species in this community, how rates of molt‐breeding overlap influence individual survival and productivity, and how these interact with density‐dependent processes such as population recruitment, adult mortality, and food limitations to drive evolutionary adaptations (e.g., Ricklefs 2000). In this regard, further research examining inter‐annual variability of molt‐breeding overlap rates (at the individual and population levels) in relation to rainfall/invertebrate‐abundance (and variation in El Niño‐La Niña climate fluctuations), improved estimates of bird species' diet richness and niche‐widths, and estimates of productivity and survival would produce more comprehensive understandings about the evolutionary processes shaping the unique ecologies and life‐history characteristics of small‐island birds.

## Author Contributions

**Christopher C. De Ruyck:** conceptualization (lead), data curation (lead), formal analysis (lead), investigation (lead), methodology (lead), project administration (lead), resources (supporting), visualization (lead), writing – original draft (lead), writing – review and editing (lead). **Nicola Koper:** funding acquisition (lead), supervision (lead), writing – review and editing (supporting).

## Ethics Statement

All data were gathered using protocols listed in project F15‐026, UM project number 38891, and approved by the University of Manitoba's Animal Care Committee.

## Conflicts of Interest

The authors declare no conflicts of interest.

## Supporting information


Data S1.

## Data Availability

Data and/or code are provided as private‐for‐peer review via the following link: [https://figshare.com/s/02043b58778177abf291].
