# Supplementary material for: Ecological Drivers of Molt‐Breeding Overlap, an Unusual Life‐History Strategy of Small‐Island Birds?
Source: Ecol Evol. 2025 Jan 16;15(1):e70607. doi: 10.1002/ece3.70607 (PMC11738649; doi:10.1002/ece3.70607)
Supplement: Supplementary file 1 — Data S1. [file ECE3-15-e70607-s001.docx]

APPENDICES

Appendix S1.

Journal: Ecology and Evolution

Title: Ecological drivers of moult-breeding overlap, an unusual life-history strategy of small-island birds?

**Authors:** Christopher C. De Ruyck & Nicola Koper


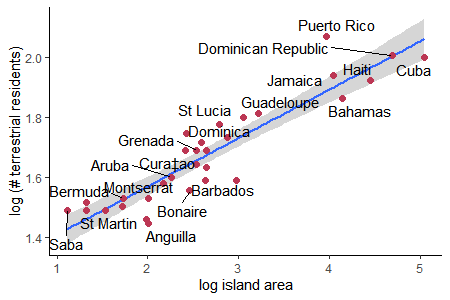


Appendix S1 - Figure S1. Log (*n* terrestrial resident bird species; Gerbracht and Levesque 2019) vs. log (island area) for 21 island-groups in the West Indies. Grenada (312 km2) sits just below 50^th^ percentile of species richness, as well as *n* species per km^2^.

##### Appendix S2.

Journal: Ecology and Evolution

Title: Ecological drivers of moult-breeding overlap, an unusual life-history strategy of small-island birds?

**Authors:** Christopher C. De Ruyck & Nicola Koper


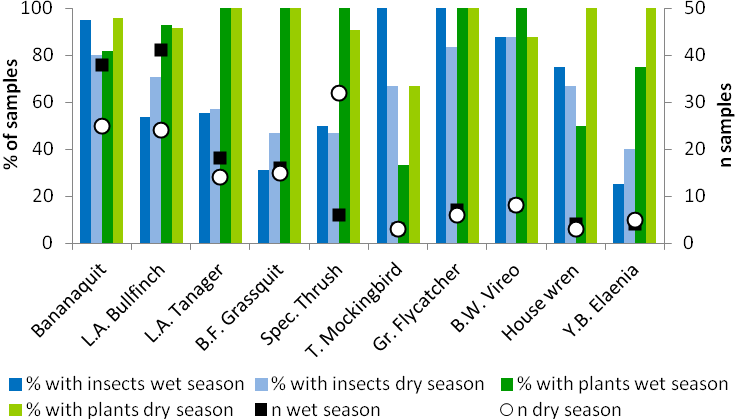


##### Appendix S2 – Figure S1. Percentage of successfully sequenced samples yielding invertebrate or plant DNA by season, Grenada, 2018-2019. Blue bars = % samples with invertebrate DNA. Green bars = % samples with plant DNA. Black squares (wet season) and white circles (dry season) = *n* samples yielding identifiable DNA (right axis).

##### Appendix S3.

Journal: Ecology and Evolution

Title: Ecological drivers of moult-breeding overlap, an unusual life-history strategy of small-island birds?

**Authors:** Christopher C. De Ruyck & Nicola Koper


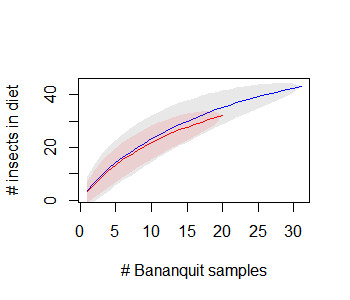

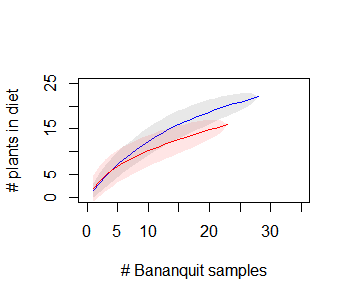


**Insects Plants**


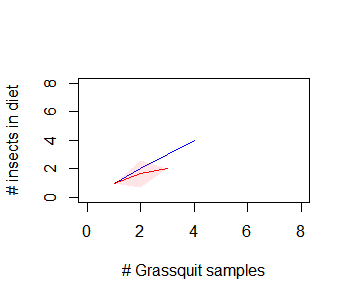

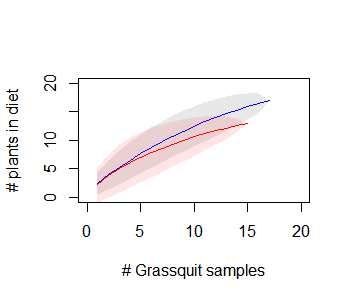


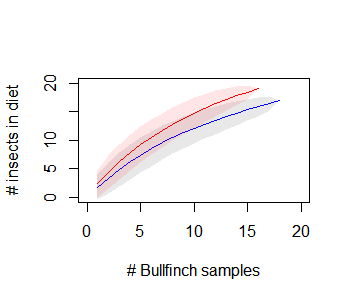

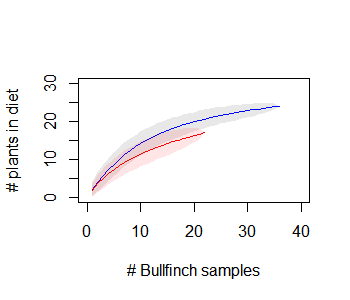


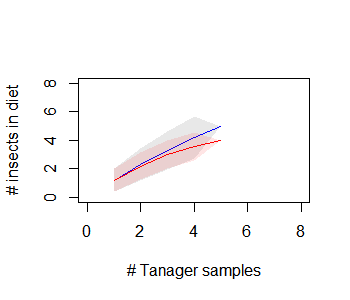

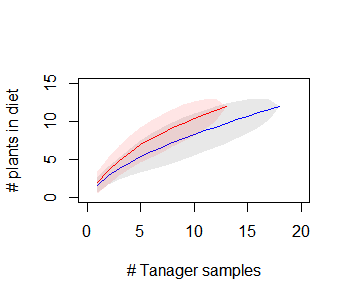


**Insects Plants**


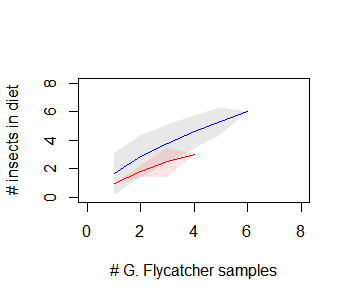

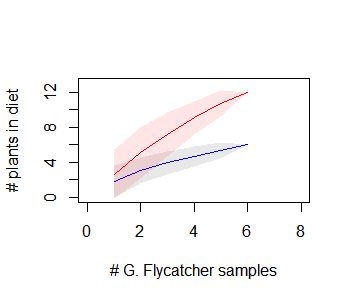


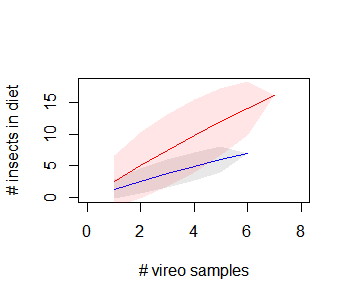

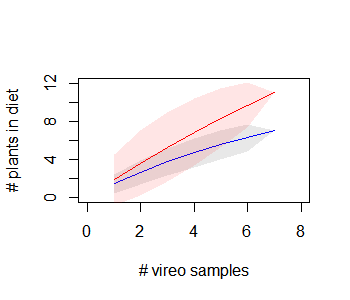


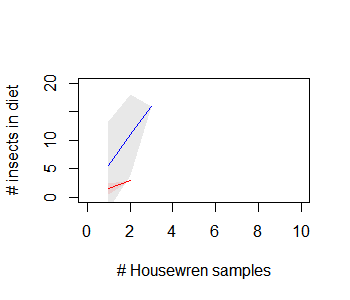

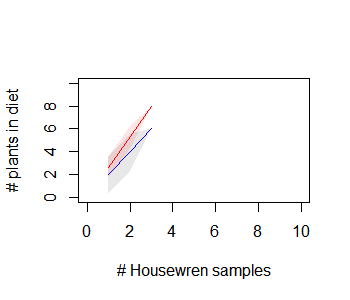


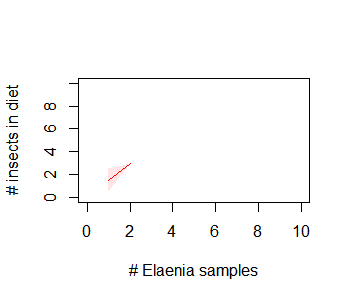

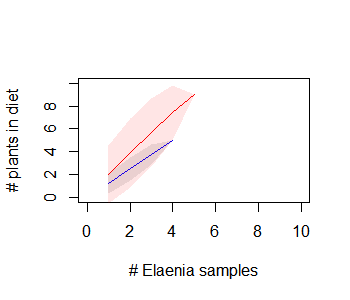


##### Appendix S3 – Figure S1. Rarefaction curves depicting accumulation of unique diet genera across 6 sites in 8 bird species with 95% confidence intervals, wet season in blue, dry season in red, invertebrates genera on left, plant genera on right, Grenada 2018-2019. Yellow-bellied Elaenia sample size = 1 for wet season invertebrates and thus no accumulation.
